# The additive effect of metabolic syndrome on left ventricular impairment in patients with obstructive coronary artery disease assessed by 3.0 T cardiac magnetic resonance feature tracking

**DOI:** 10.1186/s12933-024-02225-y

**Published:** 2024-04-23

**Authors:** Chen-Yan Min, Yue Gao, Yi-Ning Jiang, Ying-Kun Guo, Ke Shi, Zhi‑Gang Yang, Yuan Li

**Affiliations:** 1 Department of Radiology, West China Hospital, Sichuan University, 37# Guo Xue Xiang, Chengdu, 610041 Sichuan China; 2 Key Laboratory of Obstetric & Gynecologic and Pediatric Diseases and Birth Defects of Ministry of Education, Department of Radiology, West China Second University Hospital, Sichuan University, 20# Section 3, Renmin South Road, Chengdu, 610041 Sichuan China

**Keywords:** Metabolic syndrome, Obstructive coronary artery disease, Strain, Cardiac magnetic resonance

## Abstract

**Background:**

Metabolic syndrome (MetS) can increase the risk of morbidity and mortality of cardiovascular disease and obstructive coronary artery disease (OCAD), which usually have a poor prognosis. This study aimed to explore the impact of MetS on left ventricular (LV) deformation and function in OCAD patients and investigate the independent factors of impaired LV function and deformation.

**Materials and methods:**

A total of 121 patients with OCAD and 52 sex- and age-matched controls who underwent cardiac magnetic resonance scanning were enrolled in the study. All OCAD patients were divided into two groups: OCAD with MetS [OCAD(MetS+), n = 83] and OCAD without MetS [OCAD(MetS−), n = 38]. LV functional and global strain parameters were measured and compared among the three groups. Multivariable linear regression analyses were constructed to investigate the independent factors of LV impairment in OCAD patients. Logistic regression analysis and receiver operating characteristic (ROC) curve analysis were performed to test the prediction efficiency of MetS for LV impairment.

**Results:**

From controls to the OCAD(MetS−) group to the OCAD(MetS+) group, LV mass (LVM) increased, and LV global function index (LVGFI) and LV global longitudinal peak strain (GLPS) decreased (all *p* < 0.05). Compared with the OCAD(MetS−) group, the LV GLPS declined significantly (*p* = 0.027), the LVM increased (*p* = 0.006), and the LVGFI decreased (*p* = 0.043) in the OCAD(MetS+) group. After adjustment for covariates in OCAD patients, MetS was an independent factor of decreased LV GLPS (β = − 0.211, *p* = 0.002) and increased LVM (β = 0.221, *p* = 0.003). The logistic multivariable regression analysis and ROC analysis showed that combined MetS improved the efficiency of predicting LV GLPS reduction (AUC = 0.88) and LVM (AUC = 0.89) increase.

**Conclusions:**

MetS aggravated the damage of LV deformation and function in OCAD patients and was independently associated with LV deformation and impaired LV strain. Additionally, MetS increased the prediction efficiency of increased LVM and decreased LV GLPS. Early detection and intervention of MetS in patients with OCAD is of great significance.

**Supplementary Information:**

The online version contains supplementary material available at 10.1186/s12933-024-02225-y.

## Introduction

Metabolic syndrome (MetS) is a syndrome composed of various risk factors. The mechanism of myocardial injury caused by MetS is very complex and is usually related to insulin resistance, systemic inflammation, oxidative stress, and adipose tissue dysfunction [1]. Obstructive coronary artery disease (OCAD) is one of the leading causes of heart failure (CVD), a leading cause of death worldwide, and coronary angiography (CAG) can accurately identify OCAD [2]. With the aging of the population, the incidence and mortality of OCAD are increasing, which results in a burden on public health due to increased medical resources [3]. MetS has a global epidemic trend and a high incidence, which is associated with an increased risk of morbidity and mortality of OCAD, and studies have demonstrated that patients with MetS had a higher risk of OCAD than those without MetS [4–7]. Studies have found that MetS was independently associated with a higher coronary atherosclerosis burden and could lead to coronary microvascular dysfunction, while the existence of coronary microvascular dysfunction is of great significance to the prognosis of OCAD and is associated with an increased risk of adverse outcomes in patients with OCAD [8–10]. The previous studies reported that left ventricular (LV) global longitudinal peak strain (GLPS) has been associated with LV systolic dysfunction and is an effective predictor of adverse cardiovascular events, and the measurement of GLPS has been shown to improve the risk stratification of patients with heart failure [11, 12]. Therefore, it is significant to investigate the effect of MetS on LV deformation and function in patients with OCAD regarding health management.

Cardiac magnetic resonance (CMR) imaging has good spatial and tissue resolution. It can provide information on cardiac function, morphology, hemodynamics, and myocardial tissue characteristics, especially for CMR feature tracking technology, which can detect subtle cardiac morphological and functional changes early [13, 14]. A previous study has reported the effect of MetS on LV structure and function by CMR feature tracking [15]. However, the additive effect of MetS on LV deformation and function in OCAD patients remained unclear. Besides, little has been known about the study using CMR feature tracking to evaluate the additive effect of myocardial injury in MetS with OCAD patients. The purpose of this study was to investigate the additive effect of MetS on LV deformation and function in OCAD patients by CMR feature tracking and to explore the independent related factors of LV deformation and impaired LV strain.

## Methods and materials

### Study population

From January 2012 to April 2022, we retrospectively enrolled patients who were suspicious signs or symptoms of myocardial ischaemia or chest pain or suspected coronary artery disease (CAD) were selected to undergo CAG and CMR examination. These patients were further screened by the results of CAG, and patients with at least one major coronary artery stenosis ≥ 50% were defined as OCAD [16]. The interval time between CAG and CMR examination in all patients was no more than 3 months. The exclusion criteria were as follows: (1) myocardial infarction; (2) congenital heart diseases; (3) previous operation history of coronary artery revascularization; (4) primary cardiomyopathy; (5) severe cardiac arrhythmia; (6) severe valvular heart disease and (7) CMR image inadequate and poor image quality. Adhering to the definition of MetS by a joint interim statement of the International Diabetes Federation Task Force on Epidemiology and Prevention (2009) [17]. In this definition, the presence of any 3 of 5 risk factors constitutes a diagnosis of MetS: (1) elevated waist circumference (definitions based on different populations and countries); (2) elevated triglycerides [≥ 150 mg/dL (1.7 mmol/L)] or specific treatment for this lipid abnormality; (3) reduced high-density lipoprotein cholesterol [< 40 mg/dL (1.0 mmol/L) in males; < 50 mg/dL (1.3 mmol/L) in females] or specific treatment for this lipid abnormality; (4) elevated blood pressure (systolic ≥ 130 mmHg and/or diastolic ≥ 85 mmHg) or treatment of previously diagnosed hypertension; and 5) elevated fasting glucose [> 100 mg/dL (5.6 mmol/L)] or previously diagnosed type 2 diabetes mellitus (T2DM). For patients without waist circumference measurement, body mass index (BMI) was used instead of waist circumference and BMI > 25 kg/m^2^ was considered as exceeding the waist circumference threshold MetS [18]. The presence of any < 2 risk factors was defined as non-MetS.

Finally, 121 patients with OCAD were included in this study. They were divided into two groups based on whether they had MetS: OCAD with MetS [OCAD(MetS+), n = 83] and OCAD without MetS [OCAD(MetS−), n = 38]. In addition, age- and sex-matched controls who underwent CMR examination were recruited. Exclusion criteria for controls were as follows: (1) T2DM; (2) hypertension; (3) hyperlipidemia; (4) BMI ≥ 25 kg/m^2^; (5) known cardiovascular disease, and (6) abnormalities detected by CMR (abnormal ventricular motion, perfusion defect, decreased ejection fraction, valvular stenosis, etc.). A total of 52 controls were recruited in the final.

We collected the essential clinical characteristics (BMI, blood pressure, heart rate, etc.) of all subjects and the biochemical indicators and medication of patients with OCAD. The interval time between laboratory examination and CMR examination in all patients was no more than 2 weeks. Smoking was defined as current or previous smoking of at least one cigarette per day for at least 1 year [19]. T2DM was diagnosed by the American Diabetes Association guidelines [20]. According to World Health Organization BMI standards, obesity was defined as BMI ≥ 25 kg/m^2^. Hypertension was defined as systolic ≥ 140 mmHg and/or diastolic ≥ 90 mmHg measured three times on different days or a previous diagnosis of essential hypertension or usage of antihypertensive medication. The Gensini score was used to evaluate the severity of coronary artery stenosis in this study. According to the calculation method of the Gensini score [21], it was assessed by an experienced cardiologist who was blinded to the clinical and surgical data. Each OCAD patient was classified in the distribution of one, two, or three vessels by the number of diseased vessels with stenosis ≥ 50%. One vessel involvement included the left descending artery (LAD), left circumflex artery (LCX), and right coronary artery (RCA). Patients with left main coronary artery (LM) stenosis ≥ 50% were classified as having three vessel involvement [22]. The study protocol was approved by the West-China Hospital of Sichuan University Biomedical Research Ethics Committee. Written informed consent was waived because of the retrospective nature of the study.

### CMR scanning protocol

CMR examinations of all subjects were performed in the supine position using a 3.0 T whole-body magnetic resonance scanner: MAGNETOM Skyra or Tim Trio (Siemens Medical Solutions, Erlangen, Germany), with a standard ECG-triggering device and end-inspiratory breath holding. A retrospectively gated balanced steady-state free-precession (b-SSFP) sequence (repetition time [TR] 2.81/3.4 ms, echo time [TE] 1.22/1.3 ms, flip angle 38°/50°, slice thickness 8 mm, field of view 250 × 300 mm^2^ or 340 × 285 mm^2^, and matrix 208 × 139 or 256 × 166) was used to obtain cine images, which included a stack of short-axis slices from the mitral valve annulus level to the left ventricle apex, four-, and two-chamber in the long-axis views. Late gadolinium enhancement (LGE) images were acquired in the corresponding slice position as the cine imaging 10–15 min after contrast injection. The images were obtained using a phase-sensitive inversion recovery sequence with the following parameters: temporal time 300 ms, TE 1.44 ms, flip angle 40°, slice thickness 8 mm, FOV 275 × 400-mm^2^, and matrix size = 256 × 184-mm^2^.

### CMR data analysis

We uploaded all acquired image data to a semi-automated software (Cvi42; Circle Cardiovascular Imaging, Inc., Calgary, Canada). Endocardial and epicardial traces were delineated manually in serial short-axis slices during the end-diastolic and end-systolic phases by an experienced radiologist who was blinded to the clinical data. Papillary muscles were considered as part of the ventricular cavity, and epicardial fat was excluded. The LV functional parameters were automatically calculated, including LV end-diastolic volume (LVEDV), LV end-systolic volume (LVESV), LV stroke volume (LVSV), LV ejection fraction (LVEF), and LV mass (LVM). The LV global function index (LVGFI) was calculated using the following formula: LVGFI = {LVSV/[(LVEDV + LVESV)/2 + (LVM/1.05)]} × 100 [23].

**Fig. 1:**
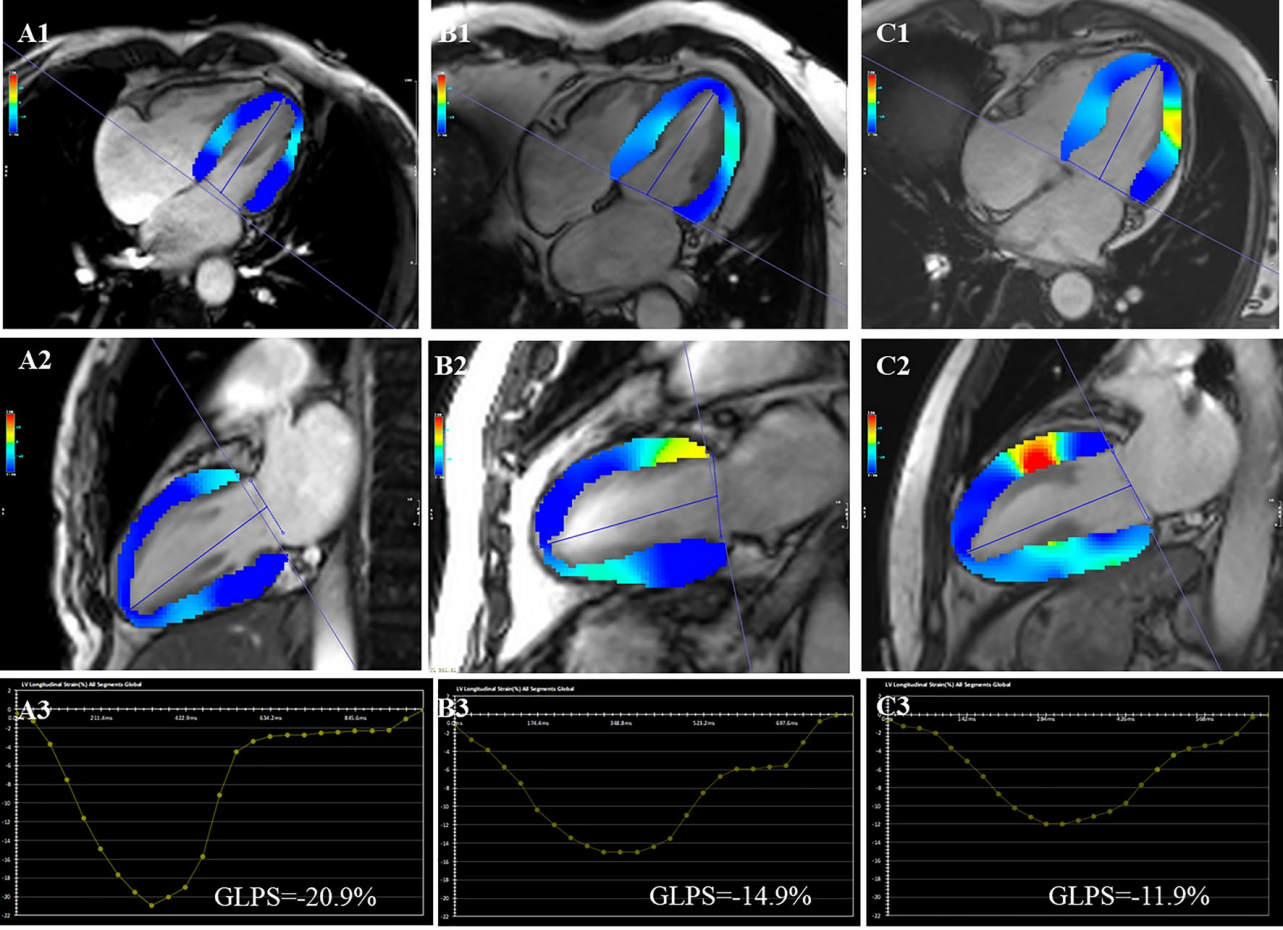
Three groups of the representative CMR imaging LV pseudo color images of long-axis four-and two-chamber cine images at the end-systole and CMR imaging derived the global longitudinal peak strain curves. **A1**–**A3** a control subject, male, 67 years, **B1**–**B3** an OCAD(MetS−) patient, male, 62 years, **C1**–**C3** an OCAD(MetS+) patient, male, 55 years

The short-axis, two- and four-chamber long-axis views were put into the feature tracking module to evaluate the LV strain. The LV global strains, such as the global radial peak strain (GRPS), global circumferential peak strain (GCPS), and GLPS, were computed by the software. The LV GRPS is positive due to myocardial thickening during LV contraction. The LV GCPS and GLPS are negative because the myocardium is shortened during contraction (Fig.1). LGE was defined as the area of signal intensity five standard deviations above the mean intensity of the normal myocardium on the LGE short axis images. Two radiologists categorized delayed enhancement into 5 categories: (1) none: in which there were no areas of LGE; (2) subendocardial: in which there were LGE is limited to subendocardial; (3) midmyocardium: in which there were LGE is limited to midmyocardium; (4) subepicardial: in which there were LGE is limited to subepicardial; (5) transmural: in which there was a whole layer, of LGE extending from the endocardium to the epicardium [24].

### Statistical analysis

Statistical analyses were performed by SPSS (version 25.0, IBM SPSS Inc., Armonk, New York, USA) and GraphPad Prism (version 9.5, GraphPad Software Inc., San Diego, CA, USA). The Shapiro–Wilk test was performed to evaluate the normality of the data, and the Levene’s test was performed to assess the homogeneity of variance of continuous variables. Continuous data were expressed as the mean ± standard deviation (SD) or median and interquartile range (IQR) according to the results of normality analysis. Categorical data were expressed as numbers (percentages). We used the Student’s t test or Mann–Whitney U test to compare the continuous variables between two groups. Baseline and cardiac MRI-related parameters were compared between the OCAD(MetS+) group, the OCAD(MetS−) group, and controls by one-way analysis of variance (one-way ANOVA), and Bonferroni’s hoc post-test or Kruskal–Wallis rank test was performed. Categorical values were compared by the chi-square test or Fisher’s exact test. Univariable analysis was performed to demonstrate the relationship between strain or statistically significant CMR parameters and MetS and other cardiovascular risk factors. To evaluate the independent related factors of the impaired LV strain and LV function in the study cohort, stepwise multivariable linear regression models were used to select variables that were not collinear in univariable analysis and had a *p* value < 0.05. Logistic regression analysis and receiver operating characteristic (ROC) curve analysis were performed to quantify the diagnostic efficiency of MetS for LV impaired strain and LV deformation. Furthermore, patients with OCAD were stratified according to median value of LV GLPS and LVM to assess impaired LV GLPS and increased LVM. The value > − 7.21% was defined as impaired LV GLPS, and the value > 115.371 g was defined as increased LVM. For all analyses, a *p* value < 0.05 was regarded as indicating statistical significance. Intraclass correlation coefficients (ICCs) were used to evaluate intra- and inter-observer reproducibility.

### Variability analysis

In order to analyze the intra-observer variability, a researcher measured LV global strain and functional parameters twice in 40 randomly selected subjects (30 OCAD patients and 10 controls), with an interval of 1 month. Then, the second researcher, who did not know the results of the first researcher, reanalysed the measurement results. Finally, the variability between observers was evaluated based on the results of the two researchers. The two researchers were independent and double-blind.

## Results

### Baseline characteristics of the study cohort

A total of 121 OCAD [OCAD(MetS−): n = 38, 76.3% males, 62 ± 12 years; OCAD(MetS+): n = 83, 79.5% males, 60 ± 10 years] patients and 52 controls (75% males, 59 ± 8 years) were enrolled in this study. The baseline characteristics of the study cohort are shown in Table 1. Compared with the controls and the OCAD(MetS−) group, BMI was higher in the OCAD(MetS+) group (both *p* < 0.001), and age and sex were no statistically significant difference. More patients in the OCAD(MetS+) group existed T2DM, HTN, and obesity than in the OCAD(MetS−) group (all *p* < 0.001). In the OCAD(MetS+) group, the proportion of LCX stenosis [52 (63.4%) vs. 13 (34.2%), *p* = 0.003] and the number of the obstructive coronary artery were higher (*p* = 0.010) than these in OCAD(MetS−) group. There was no statistically significant difference in the Gensini score, but the median had an upwards trend from the OCAD(MetS−) group to the OCAD(MetS+) group. Median triglycerides, glycated hemoglobin, glucose, and triglyceride index increased from the OCAD(MetS−) group to the OCAD(MetS+) group, while high-density lipoprotein decreased (all *p* < 0.001). In terms of medication, more patients in the OCAD(MetS+) group used oral or injected insulin, biguanides, and angiotensin-converting enzyme inhibitor/angiotensin receptor blocker (ACEI/ARB) (all *p* < 0.01).

**Table 1:** Baseline characteristics of the study cohort

	Controls (n = 52)	OCAD(MetS−) (n = 38)	OCAD(MetS+) (n = 83)	P value
Baseline characteristics				
Age (y)	$59\pm 8$	$62\pm 12$	60 $\pm 10$	0.427
Male (n, %)	39 (75.0%)	29 (76.3%)	66 (79.5%)	0.815
BMI (kg/m^2^)	$22.13\pm 1.96$	21.71 $\pm 1.92$	26.38 $\pm 2.80$^*#^	< 0.001
Heart rate (bpm)	74 (63–80)	75 (64–87)	73 (66–83)	0.615
SBP (mmHg)	$126\pm 17$	121 $\pm 27$	133 $\pm 21$^#^	0.012
DBP (mmHg)	$76\pm 10$	79 $\pm 10$	81 $\pm 13$	0.075
NYHA classification (n, %)				
I/II/III/IV	–	7 (18.5%)/14 (36.8%)/13 (34.2%)/4 (10.5%)	14 (16.9%)/40 (48.2%)/21 (25.3%)/8 (9.6%)	0.671
Cardiovascular risk factors (n, %)				
T2DM	0 (0)	2 (5.3%)	51 (61.4%)*^#^	< 0.001
HTN	0 (0)	7 (18.4%)*	67 (80.7%)*^#^	< 0.001
Obesity	0 (0)	1 (2.6%)	65 (78.3%)*^#^	< 0.001
Smoking	–	21 (55.3%)	41 (49.4%)	0.549
Heart failure	–	0 (0%)	4 (4.8%)	0.407
Moderate valvular heart disease	–	3 (7.9%)	6 (7.2%)	1.000
Coronary related parameters				
Gensini score	–	24.75 (11.75–82.00)	49.25 (20.88–96.25)	0.196
Location of the obstructive coronary artery (n, %)				
LM	–	4 (10.5%)	7 (8.5%)	0.991
LAD	–	29 (76.3%)	72 (87.8%)	0.109
LCX	–	13 (34.2%)	52 (63.4%)^#^	0.003
RCA	–	17 (44.7%)	51 (61.4%)	0.086
Number of the obstructive coronary artery (n, %)				
(One/Two/Three-vessel)	–	24 (63.2%)/5 (13.2%)/9 (23.6%)	29 (35.4%)/11 (13.4%)/42 (51.2%)	0.010
Laboratory parameters				
TG (mmol/l)	–	1.05 (0.79–1.51)	1.87 (1.33–2.67)^#^	< 0.001
TC (mmol/l)	–	3.86 (3.15–4.84)	3.45 (2.91–4.35)	0.065
HDL (mmol/l)	–	1.30 (1.10–1.44)	0.89 (0.77–1.00)^#^	< 0.001
LDL (mmol/l)	–	2.08 (1.50–2.85)	1.85 (1.48–2.41)	0.182
HbA1c (%)	–	6.16 (5.88–6.23)	6.91 (6.30–6.93)^#^	< 0.001
GLU (mmol/l)	–	5.25 (4.74–6.67)	6.88 (5.55–8.76)^#^	< 0.001
Triglyceride index	–	3.07 (2.16–4.78)	6.75 (3.94–12.54)^#^	< 0.001
NT-proBNP (pg/mL)	–	311.00 (69.50–1568.50)	510.00 (117.00–1774.00)	0.350
eGFR (mL/min/1.73 m^2^)	–	81.69 (72.39–91.96)	82.44 (72.67–94.14)	0.876
Medication (n, %)				
SGLT2 inhibitors	–	1 (2.6%)	5 (6.0%)	0.729
Insulin	–	1 (2.6%)	22 (26.5%)^#^	0.002
Biguanides	–	0 (0)	23 (27.7%)^#^	< 0.001
ACEI/ARB	–	14 (36.8%)	61 (73.5%)^#^	< 0.001
Beta-blocker	–	25 (65.8%)	58 (69.9%)	0.653
Satins	–	35 (92.1%)	77 (92.8%)	0.897
Anti-thrombotic agents	–	34 (89.5%)	79 (95.2%)	0.241

Data are presented as the mean ± SD, median (Q1–Q3) or number (percentage)

*OCAD* coronary artery disease, *MetS* metabolic syndrome, *BMI* body mass index, *SBP* systolic blood pressure, *DBP* diastolic blood pressure, *T2DM* type 2 diabetes mellitus, *HTN* hypertension, *LM* left main coronary artery, *LAD* left descending artery, *LCX* left circumflex artery, *RCA* right coronary artery, *TG* triglycerides, *TC* triglyceride, *HDL* high-density lipoprotein cholesterol, *LDL* low-density lipoprotein cholesterol, *HbA1c* glycated hemoglobin, *Glu* glucose, *eGFR* estimated glomerular filtration rate, *NT-proBNP* amino-terminal pro-B-type natriuretic peptide, *ACEI* angiotensin-converting enzyme inhibitor, *ARB* angiotensin receptor blocker, *SGLT2* sodium-dependent glucose transporters 2

*P less than 0.05 vs. the controls group

^#^P less than 0.05 vs. the OCAD(MetS−) group

### Comparison of LV functional and strain parameters among OCAD patients with and without MetS and controls

**Fig. 2:**
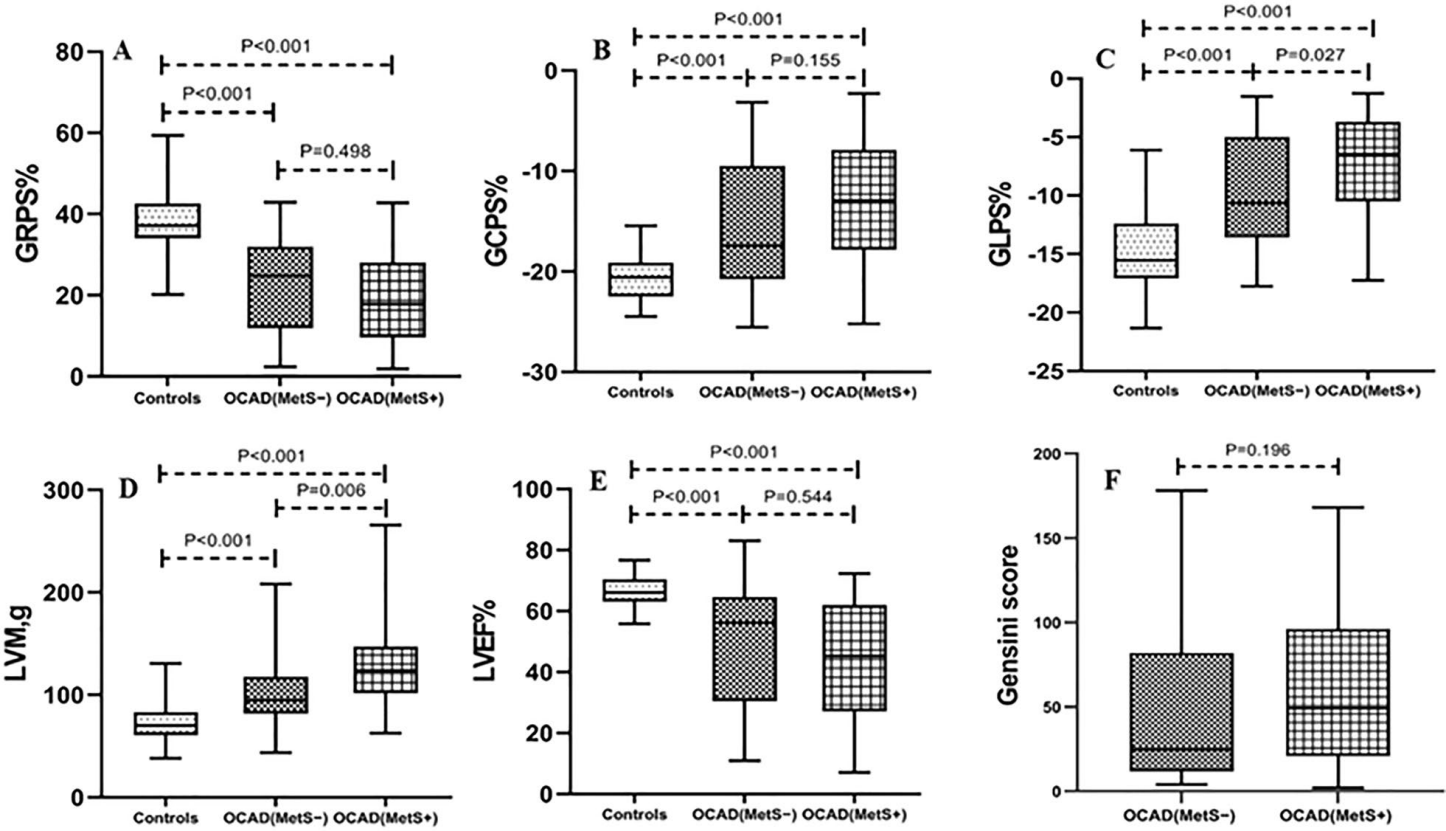
Comparison of the LV function and global strain among three groups, and the Gensini score between the OCAD(MetS−) group and the OCAD(MetS+) group. *LVM* left ventricular mass, *LVEF* left ventricular ejection fraction, *GRPS* global radial peak strain, *GCPS* global circumferential peak strain, *GLPS* global longitudinal peak strain

The LV functional and strain parameters are presented in Table 2. From controls to the OCAD(MetS−) group to the OCAD(MetS+) group, the LVM increased and LVGFI decreased (all *p* < 0.05). The LVM [94.73 (81.55–117.94) g vs. 123.79 (101.53–147.21) g, *p* = 0.006] increased significantly, and the LVGFI (37.14 ± 16.51 vs. 30.99 ± 13.16, *p* = 0.043) decreased in the OCAD(MetS+) group compared with the OCAD(MetS−) group. The LV GLPS decreased from controls to the OCAD(MetS−) group to the OCAD(MetS+) group (all p < 0.05). Compared with the OCAD(MetS−) group, the LV GLPS reduced significantly in the OCAD(MetS+) group [− 10.63 (4.98–13.57) % vs. − 6.49 (3.77–10.70) %, *p* = 0.027]. The LV GRPS and GCPS were lower in the OCAD(MetS+) and OCAD(MetS−) groups compared with the controls (all *p* < 0.001). Compared with the OCAD(MetS−) group, The LV GRPS and GCPS [radial: OCAD(MetS−) vs. OCAD(MetS+): 24.76 (11.93–31.98)% vs. 17.85 (9.65–28.09)%, *p* = 0.498; circumferential: OCAD(MetS−) vs. OCAD(MetS+): − 17.46 (9.47–20.78)% vs. − 12.93 (7.88–17.83)%, *p* = 0.155] showed a downwards trend, whereas there was no statistically significant difference in myocardial strain between the two groups (Fig. 2). LV LGE was evaluated in 112 OCAD patients who underwent LGE examination [(OCAD(MetS−): n = 36; OCAD(MetS+): n = 76]. 49 patients with OCAD had LGE, of which 25 patients had LGE type of subendocardial, 13 patients with midmyocardium LGE, 2 patients with subepicardial LGE, and 9 patients with transmural LGE, and the results are presented in Table 2.

**Table 2:** CMR parameters among OCAD patients with and without MetS and controls

	Controls (n = 52)	OCAD(MetS−) (n = 38)	OCAD(MetS+) (n = 83)	P value
Function parameters				
LVEDV (mL)	120.19 (105.81–138.65)	142.85 (118.10–212.32)*	176.42 (145.77–263.82)*	< 0.001
LVESV (mL)	39.64 (33.01–48.81)	60.38 (42.79–152.79)*	93.12 (53.35–192.49)*	< 0.001
LVSV (mL)	$81.48\pm 17.78$	$73.22\pm 17.17$	$78.23\pm 23.87$	0.181
LVEF (%)	66.02 (63.05–70.42)	56.12 (30.43–64.64)*	45.27 (27.07–61.97)*	< 0.001
LVM (g)	70.06 (60.96–83.23)	94.73 (81.55–117.94)*	123.79 (101.53–147.21)*^#^	< 0.001
LVGFI	53.99 $\pm$ 7.94	37.14 $\pm 16.51$*	30.99 $\pm 13.16$*^#^	< 0.001
LV strain parameters				
GRPS (%)	37.18 (34.02–42.66)	24.76 (11.93–31.98)*	17.85 (9.65–28.09)*	< 0.001
GCPS (%)	− 20.59 (19.10–22.48)	− 17.46 (9.47–20.78)*	− 12.93 (7.88–17.83)*	< 0.001
GLPS (%)	− 15.53 (12.38–17.07)	− 10.63 (4.98–13.57)*	− 6.49 (3.77–10.70)*^#^	< 0.001

LGE pattern		OCAD(MetS−) (n = 36)	OCAD(MetS+) (n = 76)	
None, n (%)	–	23 (63.9%)	40 (52.6%)	0.262
Subendocardial, n (%)	–	7 (19.4%)	18 (23.7%)	0.615
Midmyocardium, n (%)	–	2 (5.6%)	11 (14.5%)	0.289
Subepicardial, n (%)	–	1 (2.8%)	1 (1.3%)	1.000
Transmural, n (%)	–	3 (8.3%)	6 (7.9%)	1.000

Data are presented as the mean ± SD, median (Q1–Q3)

*OCAD* coronary artery disease, *MetS* metabolic syndrome, *LVEDV* left ventricular end-diastolic volume, *LVESV* left ventricular end-systolic volume, *LVSV* left ventricular stroke volume, *LVEF* left ventricular ejection fraction, *LVM* left ventricular mass, *LVGFI* left ventricular global function index, *GRPS* global radial peak strain, *GCPS* global circumferential peak strain, *GLPS* global longitudinal peak strain, *LGE* late gadolinium enhancement

*P less than 0.05 vs. the controls group

^#^P less than 0.05 vs. the OCAD(MetS−) group

### Association between clinical risk factors and impaired LV strain and deformation in OCAD patients

The univariable analyses in OCAD patients exhibited that MetS had a negative correlation with the LV GLPS (β = − 0.273, *p* = 0.002) and LVGFI (β = − 0.198, *p* = 0.030) and a positive correlation with LVM (β = 0.268, *p* = 0.003). In addition, amino-terminal pro-B-type natriuretic peptide (NT-proBNP), Gensini score, smoking, and ACEI/ARB were common variables associated with the LV GLPS, LVGFI, and LVM (all p < 0.05) (Table 3). After multivariable adjustment, MetS was an independent factor of decreased LV GLPS (β = − 0.211, *p* = 0.002) and increased LVM (β = 0.221, *p* = 0.003). In addition, NT-proBNP also had an independent correlation with impaired LV GLPS (β = − 0.556, p < 0.001) and increased LVM (β = 0.395, p < 0.001) (Table 3).

**Table 3:** Determinants of impaired LV function and deformation in OCAD patients

	GLPS				LVGFI				LVM			
	Univariable		Multivariable		Univariable		Multivariable		Univariable		Multivariable	
	β	P value	β	P value	β	P value	β	P value	β	P value	β	P value
MetS	− 0.273	0.002	− 0.211	0.002	− 0.198	0.030	− 0.065	0.345	0.268	0.003	0.221	0.003
Male (n)	− 0.183	0.045	− 0.070	0.367	− 0.199	0.029	− 0.174	0.017	0.410	< 0.001	0.336	< 0.001
Age (y)	− 0.029	0.751			− 0.023	0.800			− 0.174	0.057		
Smoking	− 0.264	0.003	− 0.215	0.001	− 0.259	0.004	− 0.148	0.043	0.346	< 0.001	0.165	0.047
HbA1c (%)	− 0.294	0.001	− 0.058	0.436	− 0.357	< 0.001	− 0.195	0.004	0.088	0.335		
eGFR (mL/min/1.73 m^2^)	0.117	0.202			0.153	0.094			− 0.043	0.637		
NT-proBNP^a^ (pg/mL)	− 0.629	< 0.001	− 0.556	< 0.001	− 0.670	< 0.001	− 0.609	< 0.001	0.412	< 0.001	0.395	< 0.001
Gensini score	− 0.346	< 0.001	− 0.164	0.016	− 0.291	0.001	− 0.084	0.208	0.183	0.045	0.045	0.553
ACEI/ARB	− 0.311	0.001	− 0.107	0.132	− 0.200	0.028	− 0.032	0.619	0.193	0.034	0.026	0.740

β is the adjusted regression coefficient

Abbreviations as listed in Tables 1 and 2

^a^NT-proBNP was log-transformed before being included in the regression analysis

**Fig. 3:**
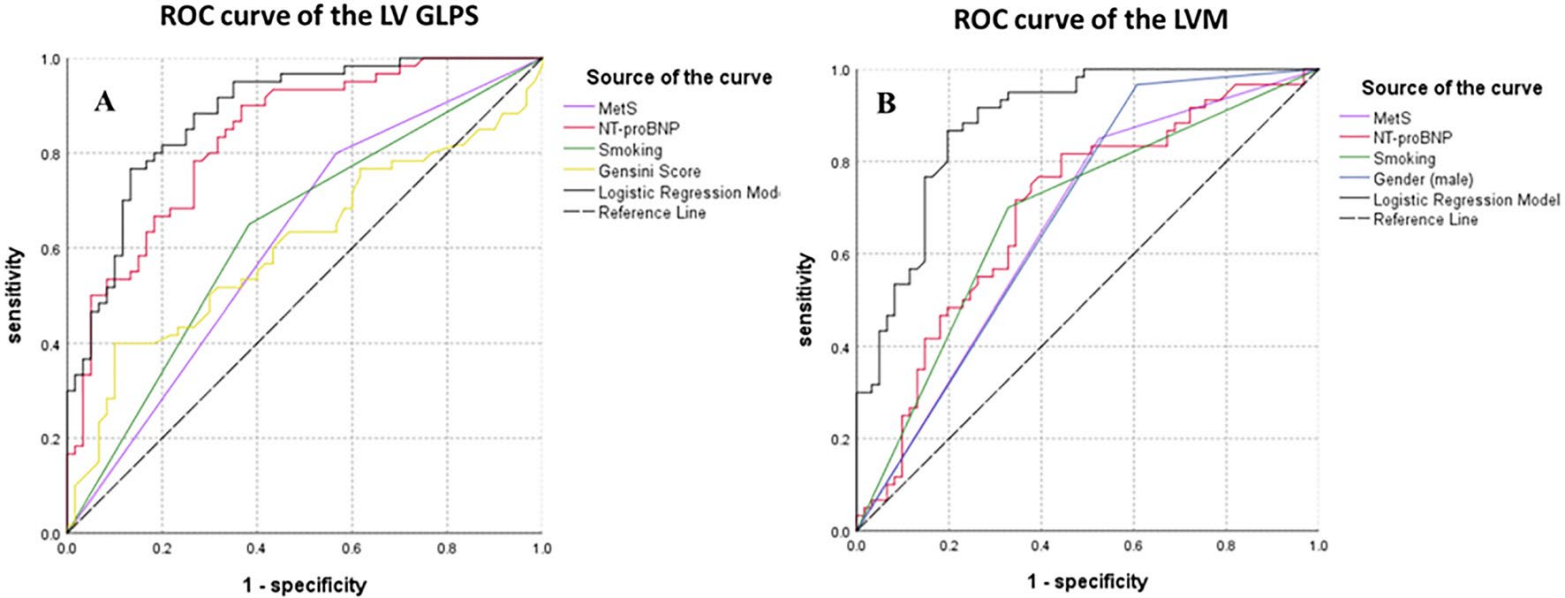
Receiver operating characteristics curve to evaluate the prediction efficiency of decreased LV GLPS (**A**) and increased LVM (**B**). The explanation of NT-proBNP and logistic regression model are listed in Table 5

On the other hand, logistic multivariable regression analysis demonstrated that MetS had independent predictive efficacy of impaired LV GLPS [OR 4.32, 95% CI (1.44–12.94), *p* = 0.009] and increased LVM [OR 10.68, 95% CI (3.31–34.45), *p* < 0.001] (Table 4). The results of the ROC analysis are shown in Table 5. The multi-parameter combination, including MetS, NT-proBNP, smoking, and Gensini score showed a sensitivity of 81.7% and specificity of 78.3% to predict the decreased LV GLPS [AUC = 0.88; 95% CI (0.83–0.94)]. The combination of MetS, NT-proBNP, smoking, and gender (male) predicted increased LVM [AUC = 0.89; 95% CI (0.83–0.94)], the sensitivity was 85.0%, and the specificity was 80.0% (Fig. 3).

**Table 4:** Multivariable logistic regression analysis of LV function and deformation in OCAD patients

	GLPS		LVM	
	OR (95% CI)	P value	OR (95% CI)	P value
MetS	4.32 (1.44–12.94)	0.009	10.68 (3.31–34.45)	< 0.001
Smoking	4.61 (1.67–12.79)	0.003	3.11 (1.04–9.31)	0.042
NT-proBNP^a^ (pg/mL)	10.34 (4.24–25.17)	< 0.001	4.16 (1.96–8.85)	< 0.001
Gensini score	1.00 (0.99–1.01)	0.655	–	–
Gender (male, n)	–	–	28.41 (4.36–185.35)	< 0.001

*LVM* left ventricular mass, *GLPS* global longitudinal peak strain, *OR* odds ratio, *MetS* metabolic syndrome, *NT-proBNP* amino-terminal pro-B-type natriuretic peptide

^a^NT-proBNP was log-transformed before being included in the regression analysis

**Table 5:** Receiver operating characteristic curve of LV function and deformation in OCAD patients

	GLPS					LVM				
	AUC% (95% CI)	Sensitivity%	Specificity%	PPV%	NPV%	AUC% (95% CI)	Sensitivity%	Specificity%	PPV%	NPV%
MetS	0.62 (0.52–0.72)	80.0	43.3	58.5	68.4	0.67 (0.57–0.76)	85.0	48.3	62.1	76.3
NT-proBNP^a^ (pg/mL)	0.83 (0.76–0.91)	71.7	73.3	72.8	72.1	0.70 (0.60–0.80)	65.0	66.7	66.1	65.5
Smoking	0.63 (0.53–0.73)	65.0	61.7	62.9	63.7	0.68 (0.59–0.78)	70.0	66.7	67.7	68.9
Gensini score	0.61 (0.50–0.71)	51.7	66.7	60.7	57.9	–	–	–	–	–
Gender (male, n)	–	–	–	–	–	0.68 (0.58–0.77)	96.7	38.3	61.0	80.0
Logistic regression model^b^	0.88 (0.83–0.94)	81.7	78.3	79.0	81.0	0.89 (0.83–0.94)	85.0	80.0	80.9	84.2

*LVM* left ventricular mass, *GLPS* global longitudinal peak strain, *AUC* area under curve, *MetS* metabolic syndrome, *NT-proBNP* amino-terminal pro-B-type natriuretic peptide, *PPV* positive predictive value, *NPV* negative predictive value

^a^NT-proBNP was log-transformed before being included in the regression analysis

^b^The model combined MetS, NT-proBNP, smoking, and Gensini score in GLPS; the model combined MetS, NT-proBNP, smoking, and Gender (male) in LVM

When we excluded 11 individuals who had heart failure or moderate valvular heart disease, the associations did not change substantially in the adjusted model. The statistically differences were significant for the LV GLPS, LVM, and LVGFI from controls to the OCAD(MetS−) group to the OCAD(MetS+) group (all p < 0.05). MetS was still an independent factor of decreased LV GLPS (β = − 0.236, p = 0.001) and increased LVM (β = 0.255, p = 0.001) (Additional file 1: Table S1). And combined MetS improved the efficiency of predicting LV GLPS reduction [AUC = 0.91; 95% CI (0.86–0.96)] and LVM [AUC = 0.92; 95% CI (0.86–0.97)] increase (Additional file 1: Tables S2, S3).

### Intra‑ and inter-observer variability

There was excellent intra- and inter-observer agreement in terms of LV function and global strain. The intra- and inter-observer agreement was excellent for LV GLPS (ICC = 0.936–0.982 and 0.903–0.972, respectively) and LVM (ICC = 0.970–0.991 and 0.977–0.994, respectively). The ICCs for intra-observer variability of LV GCPS, LV GRPS, LVEDV, and LVESV were 0.972–0.992, 0.963–0.989, 0.948–0.985, and 0.987–0.996, respectively. The ICCs for inter-observer variability of those LV strain and function parameters were 0.937–0.982, 0.967–0.991, 0.979–0.994, and 0.992–0.998, respectively (Additional file 2).

## Discussion

This study investigated the combined effect of MetS on LV deformation and function in patients with OCAD by CMR feature tracking. The main findings of this study were as follows: (1) MetS aggravated LV deformation and functional damage in OCAD patients, especially the LV GLPS; (2) for OCAD patients, MetS was found to be an independent factor of impaired LV GLPS and increased LVM; (3) the ROC analysis suggested that MetS increased the prediction efficiency of decreased LV GLPS and increased LVM; (4) the degree of coronary stenosis was higher in patients with OCAD comorbid with MetS; and (5) the impaired LV GLPS and increased LVM also had an independent correlation with NT-proBNP in OCAD patients.

### The additive effect of LV longitudinal dysfunction in patients with OCAD comorbid with MetS

Previous studies have confirmed impaired longitudinal myocardial strain in patients with MetS alone [25, 26]. For OCAD patients, our study found that MetS led to a further reduction of the LV GLPS, and MetS was independently associated with impaired LV GLPS in OCAD patients. Besides, MetS increased the prediction efficiency of impaired LV GLPS. There have been few studies on this additive effect recently, and the comprehensive mechanism of myocardial injury is still unclear. The possible potential mechanism of the additive effect is as follows: myocardial injury in OCAD patients with MetS may be related to the aggravation of coronary microvascular dysfunction. Coronary microvascular dysfunction occurs in the early stage of MetS [27]. In MetS, insulin resistance and abnormal deposition of adipose tissue increase oxidative stress and inflammatory response, thereby impairing endothelial function and reducing the bioavailability of nitric oxide [28, 29]. In addition, the activation of the renin–angiotensin–aldosterone system and increased free fatty acids in MetS result in coronary circulation vasoconstriction [30]. Hence, increased vasoconstriction and decreased vasodilator substances together lead to coronary microvascular dysfunction, resulting in myocardial ischemia. When MetS is combined with OCAD, we speculate that this coronary microvascular dysfunction may be amplified, promoting the aggravation of myocardial dysfunction. Subendocardial fibers are most vulnerable to the adverse effect of coronary microvascular dysfunction [31]. Longitudinal myocardial strain is associated with subendocardial fibers. Therefore, the damage of LV GLPS usually occurs first. This can partly explain the additive effect and the comprehensive factors behind it need to be further studied.

### MetS aggravated LV deformation and remodeling in OCAD patients

Presently, some studies have reported that the LVM increased in MetS patients [32, 33]. In OCAD patients, our study demonstrated that the combination of MetS increased LVM and decreased LVGFI, and MetS was an independent factor of increased LVM. Besides, combined with MetS, the prediction efficiency of increased LVM enhanced. LVGFI is a relatively reliable integrated LV function index, which reflects different degrees of structural LV remodeling [23]. The result may be mainly associated with MetS-induced oxidative stress. One study has shown that mitochondrial dysfunction oxidative stress affected myocardial and vascular remodeling [34]. In MetS, mitochondrial oxidative stress is mainly related to excessive reactive oxygen species, mitochondrial-dependent cardiac pump dysfunction, and cardiomyocyte apoptosis, which causes irreversible structural and functional damage to cardiomyocytes, leading to cardiac hypertrophy and remodeling [35, 36]. For MetS with OCAD patients, mitochondrial abnormal oxidative stress leads to insufficient myocardial energy supply, which further aggravates myocardial ischemia and hypoxia, leading to myocardial deformation and remodeling.

Besides, this study showed that NT-proBNP levels were independently associated with increased LVM and impaired LV GLPS in OCAD patients. NT-proBNP was usually secreted when atrial and ventricular pressure and volume load increased, but its increased expression was also associated with myocardial ischemia and hypoxia [37], which confirmed that NT-proBNP levels increased in patients with OCAD. Previous studies have found that the NT-proBNP concentration was related to the prognosis and risk stratification of patients with CAD [38]. Our finding may reflect the prognostic value of LV GLPS and LVM similar to NT-proBNP in OCAD patients.

### The degree of coronary stenosis increased in OCAD patients with MetS

Our study found that the degree of coronary stenosis in OCAD patients with MetS was more severe than in OCAD patients without MetS. In this study, the median of Gensini score was higher in OCAD patients with MetS than that of patients without MetS. Besides, the OCAD patients with MetS had significant coronary stenosis and more vessel involvement, and more patients have LCX stenosis and three vessel involvement compared with OCAD patients without MetS. Previous studies reported consistent results. A prior study has identified that MetS reduced the collateral circulation of chronic coronary occlusion [39]. A large-scale study has found that MetS patients had more severe coronary stenosis and multiple vascular involvement [40]. Insulin resistance is significantly associated with the development and progression of coronary atherosclerosis, and adverse plaque characteristics [41], which may be the possible mechanism of coronary stenosis increased in MetS patients. Gensini score is one of the widely used angiographic scoring systems for quantifying the severity of CAD [21]. Currently, study reported that Gensini score combined with some biochemical indicators can predict long-term outcomes in CAD [42]. Studies have showed that more severe coronary stenosis has higher rates of progression to occlusion and myocardial infarction, and the number of coronary vessel involvement is also predictive of survival in patients with CAD [43]. Therefore, the management of MetS is of great significance for patients with OCAD. Early diagnosis and intervention of MetS may delay the progression of coronary stenosis and improve the survival rate in OCAD patients. For patients with OCAD comorbid with MetS, strengthening the follow-up of the degree of coronary stenosis may guide the treatment of these patients.

## Limitations

Our study has several limitations. First, this was a retrospective and single-center study, so that some biases may influence the results. Second, for patients who didn't measure waist circumference, BMI was used to instead waist circumference. The acquisition of BMI was more accurate and convenient than waist circumference. A previous study has confirmed the feasibility of this alternative [18]. Third, this is a retrospective study and data collection was limited. We did not assess microvascular dysfunction in the study. Finally, this study was a cross-sectional analysis, lack of information on the impact of LV remodeling on CVD outcomes in OCAD patients with and without MetS. The long-term development in OCAD patients with or without MetS should be further investigated in follow-up in the future.

## Conclusions

For patients with OCAD, MetS aggravated LV deformation and functional damage, and it was independently associated with increased LVM and decreased LV GLPS. Combination with MetS increased the ability to predict myocardial injury in OCAD patients. More attention should be given to the early screening and intervention treatment of patients with OCAD combined with MetS to delay the progression of myocardial damage and improve the prognosis of these patients.

### Supplementary Information

**Additional file 1: Table S1.** Determinants of LV impairment in OCAD patients with excluded heart failure or moderate valvular heart disease. **Table S2.** Multivariable logistic regression analysis of LV function and deformation in OCAD patients with excluded heart failure or moderate valvular heart disease. **Table S3.** Receiver operating characteristic curve of LV function and deformation in OCAD patients with excluded heart failure or moderate valvular heart disease.

**Additional file 2: Table S4.** Inter- and intra-observer variability of CMR parameters.

## Data Availability

The datasets used and analyzed during the current study are available from the corresponding author on reasonable request.

## Abbreviations

MetS: Metabolic syndrome
CAD: Coronary artery disease
OCAD: Obstructive coronary artery disease
CVD: Cardiovascular disease
LV: Left ventricular
CMR: Cardiac magnetic resonance
CAG: Coronary angiography
T2DM: Type 2 diabetes mellitus
BMI: Body mass index
LAD: Left descending artery
LCX: Left circumflex artery
RCA: Right coronary artery
LM: Left main coronary artery
LVEDV: Left ventricular end-diastolic volume
LVESV: Left ventricular end-systolic volume
LVSV: Left ventricular stroke volume
LVEF: Left ventricular ejection fraction
LVM: Left ventricular mass
LVGFI: Left ventricular global function index
GRPS: Global radial peak strain
GCPS: Global circumferential peak strain
GLPS: Global longitudinal peak strain
LGE: Late gadolinium enhancement
NT-proBNP: Amino-terminal pro-B-type natriuretic peptide
OR: Odds ratio
AUC: Area under curve
ROC: Receiver operating characteristics

## References

[1] Sperling LS, Mechanick JI, Neeland IJ, Herrick CJ, Després JP, Ndumele CE, Vijayaraghavan K, Handelsman Y, Puckrein GA, Araneta MR (2015). The CardioMetabolic health alliance: working toward a new care model for the metabolic syndrome. J Am Coll Cardiol.

[2] Naito R, Kasai T (2022). Obstructive coronary artery disease, a common and curable but critical comorbidity in acute decompensated heart failure. Eur J Heart Fail.

[3] Wang X, Xu W, Song Q, Zhao Z, Meng X, Xia C, Xie Y, Yang C, Jin P, Wang F (2022). Association between the triglyceride-glucose index and severity of coronary artery disease. Cardiovasc Diabetol.

[4] Hirode G, Wong RJ (2020). Trends in the prevalence of metabolic syndrome in the United States, 2011–2016. JAMA.

[5] Malik S, Wong ND, Franklin SS, Kamath TV, L'Italien GJ, Pio JR, Williams GR (2004). Impact of the metabolic syndrome on mortality from coronary heart disease, cardiovascular disease, and all causes in United States adults. Circulation.

[6] Ramezankhani A, Azizi F, Hadaegh F (2022). Gender differences in changes in metabolic syndrome status and its components and risk of cardiovascular disease: a longitudinal cohort study. Cardiovasc Diabetol.

[7] Lee HS, Kim HL, Kim MA, Oh S, Kim M, Park SM, Yoon HJ, Byun YS, Park SM, Shin MS (2020). Sex difference in the association between metabolic syndrome and obstructive coronary artery disease: analysis of data from the KoRean wOmen’s chest pain rEgistry (KoROSE). J Womens Health.

[8] Berwick ZC, Dick GM, Tune JD (2012). Heart of the matter: coronary dysfunction in metabolic syndrome. J Mol Cell Cardiol.

[9] Wong ND, Nelson JC, Granston T, Bertoni AG, Blumenthal RS, Carr JJ, Guerci A, Jacobs DR, Kronmal R, Liu K (2012). Metabolic syndrome, diabetes, and incidence and progression of coronary calcium: the multiethnic study of atherosclerosis study. JACC Cardiovasc Imaging.

[10] Sechtem U, Brown D, Godo S, Lanza GA, Shimokawa H, Sidik N (2020). Coronary microvascular dysfunction in stable ischaemic heart disease (non-obstructive coronary artery disease and obstructive coronary artery disease). Cardiovasc Res.

[11] Halliday BP, Senior R, Pennell DJ (2021). Assessing left ventricular systolic function: from ejection fraction to strain analysis. Eur Heart J.

[12] DeVore AD, McNulty S, Alenezi F, Ersboll M, Vader JM, Oh JK, Lin G, Redfield MM, Lewis G, Semigran MJ (2017). Impaired left ventricular global longitudinal strain in patients with heart failure with preserved ejection fraction: insights from the RELAX trial. Eur J Heart Fail.

[13] Kammerlander AA, Donà C, Nitsche C, Koschutnik M, Schönbauer R, Duca F, Zotter-Tufaro C, Binder C, Aschauer S, Beitzke D (2020). Feature tracking of global longitudinal strain by using cardiovascular MRI improves risk stratification in heart failure with preserved ejection fraction. Radiology.

[14] Guglielmo M, Arangalage D, Bonino MA, Angelini G, Bonanni M, Pontone G, Pascale P, Leo LA, Faletra F, Schwitter J (2023). Additional value of cardiac magnetic resonance feature tracking parameters for the evaluation of the arrhythmic risk in patients with mitral valve prolapse. J Cardiovasc Magn Reson.

[15] Huang S, Li Y, Shi K, Wang J, Jiang L, Gao Y, Yan WF, Yang ZG (2023). Impact of metabolic syndrome on left ventricular deformation and myocardial energetic efficiency compared between women and men: an MRI study. J Magn Reson Imaging.

[16] Virani SS, Newby LK, Arnold SV, Bittner V, Brewer LC, Demeter SH, Dixon DL, Fearon WF, Hess B, Johnson HM (2023). 2023 AHA/ACC/ACCP/ASPC/NLA/PCNA guideline for the management of patients with chronic coronary disease: a report of the American Heart Association/American College of Cardiology Joint Committee on clinical practice guidelines. Circulation.

[17] Alberti KG, Eckel RH, Grundy SM, Zimmet PZ, Cleeman JI, Donato KA, Fruchart JC, James WP, Loria CM, Smith SC (2009). Harmonizing the metabolic syndrome: a joint interim statement of the International Diabetes Federation Task Force on Epidemiology and Prevention; National Heart, Lung, and Blood Institute; American Heart Association; World Heart Federation; International Atherosclerosis Society; and International Association for the Study of Obesity. Circulation.

[18] Campbell DJ, Somaratne JB, Jenkins AJ, Prior DL, Yii M, Kenny JF, Newcomb AE, Schalkwijk CG, Black MJ, Kelly DJ (2011). Impact of type 2 diabetes and the metabolic syndrome on myocardial structure and microvasculature of men with coronary artery disease. Cardiovasc Diabetol.

[19] PongracBarlovic D, Harjutsalo V, Gordin D, Kallio M, Forsblom C, King G, Groop PH (2018). The association of severe diabetic retinopathy with cardiovascular outcomes in long-standing type 1 diabetes: a longitudinal follow-up. Diabetes Care.

[20] Chamberlain JJ, Rhinehart AS, Shaefer CF, Neuman A (2016). Diagnosis and management of diabetes: synopsis of the 2016 American diabetes association standards of medical care in diabetes. Ann Intern Med.

[21] Rampidis GP, Benetos G, Benz DC, Giannopoulos AA, Buechel RR (2019). A guide for Gensini score calculation. Atherosclerosis.

[22] Wang J, Yang ZG, Guo YK, Jiang Y, Yan WF, Qian WL, Fang H, Min CY, Li Y (2023). Incremental effect of coronary obstruction on myocardial microvascular dysfunction in type 2 diabetes mellitus patients evaluated by first-pass perfusion CMR study. Cardiovasc Diabetol.

[23] Mewton N, Opdahl A, Choi EY, Almeida AL, Kawel N, Wu CO, Burke GL, Liu S, Liu K, Bluemke DA (2013). Left ventricular global function index by magnetic resonance imaging—a novel marker for assessment of cardiac performance for the prediction of cardiovascular events: the multi-ethnic study of atherosclerosis. Hypertension.

[24] Boynton SJ, Geske JB, Dispenzieri A, Syed IS, Hanson TJ, Grogan M, Araoz PA (2016). LGE provides incremental prognostic information over serum biomarkers in AL cardiac amyloidosis. JACC Cardiovasc Imaging.

[25] Serrano-Ferrer J, Crendal E, Walther G, Vinet A, Dutheil F, Naughton G, Lesourd B, Chapier R, Courteix D, Obert P (2016). Effects of lifestyle intervention on left ventricular regional myocardial function in metabolic syndrome patients from the RESOLVE randomized trial. Metabolism.

[26] Burroughs Peña M, Swett K, Schneiderman N, Spevack DM, Ponce SG, Talavera GA, Kansal MM, Daviglus ML, Cai J, Hurwitz BE (2018). Cardiac structure and function with and without metabolic syndrome: the echocardiographic study of Latinos (echo-SOL). BMJ Open Diabetes Res Care.

[27] Labazi H, Trask AJ (2017). Coronary microvascular disease as an early culprit in the pathophysiology of diabetes and metabolic syndrome. Pharmacol Res.

[28] Shi M, Han S, Klier K, Fobo G, Montrone C, Yu S, Harada M, Henning AK, Friedrich N, Bahls M (2023). Identification of candidate metabolite biomarkers for metabolic syndrome and its five components in population-based human cohorts. Cardiovasc Diabetol.

[29] Liu Y, Kabakov AY, Xie A, Shi G, Singh AK, Sodha NR, Ehsan A, Usheva A, Agbortoko V, Koren G (2020). Metabolic regulation of endothelial SK channels and human coronary microvascular function. Int J Cardiol.

[30] Kosmala W, Przewlocka-Kosmala M, Szczepanik-Osadnik H, Mysiak A, O'Moore-Sullivan T, Marwick TH (2011). A randomized study of the beneficial effects of aldosterone antagonism on LV function, structure, and fibrosis markers in metabolic syndrome. JACC Cardiovasc Imaging.

[31] Camici PG, Tschöpe C, Di Carli MF, Rimoldi O, Van Linthout S (2020). Coronary microvascular dysfunction in hypertrophy and heart failure. Cardiovasc Res.

[32] Allebban Z, Gardin JM, Wong ND, Sklar SK, Bess RL, Spence MA, Pershadsingh HA (2010). Relation of metabolic syndrome components to left ventricular mass in Mexican Americans versus non-Hispanic whites. Metabolism.

[33] Burchfiel CM, Skelton TN, Andrew ME, Garrison RJ, Arnett DK, Jones DW, Taylor HA (2005). Metabolic syndrome and echocardiographic left ventricular mass in blacks: the atherosclerosis risk in communities (ARIC) study. Circulation.

[34] Murphy MP, Hartley RC (2018). Mitochondria as a therapeutic target for common pathologies. Nat Rev Drug Discov.

[35] Li A, Zheng N, Ding X (2022). Mitochondrial abnormalities: a hub in metabolic syndrome-related cardiac dysfunction caused by oxidative stress. Heart Fail Rev.

[36] Sanz RL, Inserra F, García Menéndez S, Mazzei L, Ferder L, Manucha W (2023). Metabolic syndrome and cardiac remodeling due to mitochondrial oxidative stress involving gliflozins and sirtuins. Curr Hypertens Rep.

[37] Redfors B, Chen S, Crowley A, Ben-Yehuda O, Gersh BJ, Lembo NJ, Brown WM, Banning AP, Taggart DP, Serruys PW (2018). B-type natriuretic peptide assessment in patients undergoing revascularization for left main coronary artery disease: analysis from the EXCEL trial. Circulation.

[38] Wang M, Su W, Chen H, Li H (2023). The joint association of diabetes status and NT-ProBNP with adverse cardiac outcomes in patients with non-ST-segment elevation acute coronary syndrome: a prospective cohort study. Cardiovasc Diabetol.

[39] Liu T, Wu Z, Liu J, Lv Y, Li W (2021). Metabolic syndrome and its components reduce coronary collateralization in chronic total occlusion: an observational study. Cardiovasc Diabetol.

[40] Lim S, Shin H, Lee Y, Yoon JW, Kang SM, Choi SH, Park KS, Jang HC, Choi SI, Chun EJ (2011). Effect of metabolic syndrome on coronary artery stenosis and plaque characteristics as assessed with 64-detector row cardiac CT. Radiology.

[41] Cho YR, Ann SH, Won KB, Park GM, Kim YG, Yang DH, Kang JW, Lim TH, Kim HK, Choe J (2019). Association between insulin resistance, hyperglycemia, and coronary artery disease according to the presence of diabetes. Sci Rep.

[42] Wang KY, Zheng YY, Wu TT, Ma YT, Xie X (2021). Predictive value of Gensini score in the long-term outcomes of patients with coronary artery disease who underwent PCI. Front Cardiovasc Med.

[43] Charach L, Blatt A, Jonas M, Teodorovitz N, Haberman D, Gendelman G, Grosskopf I, George J, Charach G (2021). Using the Gensini score to estimate severity of STEMI, NSTEMI, unstable angina, and anginal syndrome. Medicine.

