# Supplementary material for: The additive effect of metabolic syndrome on left ventricular impairment in patients with obstructive coronary artery disease assessed by 3.0 T cardiac magnetic resonance feature tracking
Source: Cardiovasc Diabetol. 2024 Apr 23;23:133. doi: 10.1186/s12933-024-02225-y (PMC11040951; doi:10.1186/s12933-024-02225-y)
Supplement: Supplementary file 1 — Additional file 1: Table S1. Determinants of LV impairment in OCAD patients with excluded heart failure or moderate valvular heart disease. Table S2. Multivariable logistic regression analysis of LV function and deformation in OCAD patients with excluded heart failure or moderate valvular heart disease. Table S3. Receiver operating characteristic curve of LV function and deformation in OCAD patients with excluded heart failure or moderate valvular heart disease. [file 12933_2024_2225_MOESM1_ESM.docx]

**Table S1**

**Determinants of LV impairment in OCAD patients with excluded heart failure or moderate valvular heart disease**

|  | **GLPS** | | | | | |  | | **LVGFI** | | | | | | |  | | **LVM** | | | | | | | |  |
| --- | --- | --- | --- | --- | --- | --- | --- | --- | --- | --- | --- | --- | --- | --- | --- | --- | --- | --- | --- | --- | --- | --- | --- | --- | --- | --- |
|  | **Univariable** | |  | | **Multivariable** | |  | | **Univariable** | |  | | **Multivariable** | | |  | | **Univariable** | | |  | | **Multivariable** | | | |
|  | β | P value |  | | β | P value | |  | β | P value | |  | | β | P value | |  | | β | P value | |  | | β | P value | |
| MetS | −0.310 | 0.001 | |  | −0.236 | 0.001 | |  | −0.244 | 0.010 | |  | | −0.088 | 0.227 | |  | | 0.324 | 0.001 | |  | | 0.255 | 0.001 | |
| Male(n) | −0.238 | 0.012 | |  | −0.087 | 0.273 | |  | −0.227 | 0.017 | |  | | −0.212 | 0.002 | |  | | 0.413 | <0.001 | |  | | 0.297 | <0.001 | |
| Age (y) | −0.073 | 0451 | |  |  |  | |  | −0.039 | 0.687 | |  | |  |  | |  | | −0.161 | 0.092 | |  | |  |  | |
| Smoking | −0.272 | 0.004 | |  | −0.202 | 0.003 | |  | −0.258 | 0.007 | |  | | −0.118 | 0.115 | |  | | 0.351 | <0.001 | |  | | 0.173 | 0.039 | |
| HbA1c (%) | −0.293 | 0.002 | |  | −0.024 | 0.763 | |  | −0.374 | <0.001 | |  | | −0.177 | 0.011 | |  | | 0.170 | 0.075 | |  | |  |  | |
| eGFR(ml/min/1.73 m^2^) | 0.117 | 0.224 | |  |  |  | |  | 0.161 | 0.093 | |  | |  |  | |  | | −0.061 | 0.526 | |  | |  |  | |
| NT-proBNP*(pg/mL) | −0.631 | <0.001 | |  | −0.527 | <0.001 | |  | −0.689 | <0.001 | |  | | −0.628 | <0.001 | |  | | 0.469 | <0.001 | |  | | 0.412 | <0.001 | |
| Gensini score | −0.380 | <0.001 | |  | −0.187 | 0.009 | |  | −0.327 | 0.001 | |  | | −0.097 | 0.169 | |  | | 0.224 | 0.019 | |  | | 0.012 | 0.883 | |
| ACEI/ARB | −0.304 | 0.001 | |  | −0.081 | 0.272 | |  | −0.204 | 0.033 | |  | | −0.023 | 0.740 | |  | | 0.206 | 0.031 | |  | | 0.007 | 0.926 |  |

βis the adjusted regression coefficient

Abbreviations as listed in Tables 1 and 2

* NT-proBNP was log-transformed before being included in the regression analysis

**Table S2**

**Multivariable logistic regression analysis** **of LV function and deformation in OCAD patients with excluded heart failure or moderate valvular heart disease**

|  |  | **GLPS** | |  | **LVM** | |
| --- | --- | --- | --- | --- | --- | --- |
|  |  | OR (95% CI) | P value |  | OR (95% CI) | P value |
| MetS |  | 6.71(1.90 –23.71) | 0.003 |  | 19.47(4.75–79.85) | <0.001 |
| Smoking |  | 4.59(1.50–14.03) | 0.007 |  | 4.47(1.24–16.11) | 0.022 |
| NT-proBNP*(pg/mL) |  | 12.07(4.44–32.81) | <0.001 |  | 5.06(2.11–12.16) | <0.001 |
| Gensini score |  | 1.01 (1.00–1.02) | 0.214 |  | − | − |
| Gender (male, n) |  | − | − |  | 0.023(0.002–0.248) | 0.002 |

LVM: left ventricular mass; GLPS: global longitudinal peak strain; OR: odds ratio; MetS: metabolic syndrome; NT-proBNP: amino-terminal pro-B-type natriuretic peptide.

* NT-proBNP was log-transformed before being included in the regression analysis

**Table S3**

**Receiver operating characteristic curve of LV function and deformation in OCAD patients** **with excluded heart failure or moderate valvular heart disease**

|  | **GLPS** | | | | |  | **LVM** | | | | |
| --- | --- | --- | --- | --- | --- | --- | --- | --- | --- | --- | --- |
|  | AUC% (95% CI) | Sensitivity% | Specificity% | PPV% | NPV% |  | AUC% (95% CI) | Sensitivity% | Specificity% | PPV% | NPV% |
| MetS | 0.64(0.54–0.75) | 81.8 | 45.5 | 60.0 | 71.4 |  | 0.69(0.59–0.79) | 87.3 | 50.9 | 64.0 | 80.0 |
| NT-proBNP *(pg/mL) | 0.85(0.78–0.92) | 74.5 | 76.4 | 75.9 | 75.0 |  | 0.73(0.63–0.82) | 69.1 | 69.1 | 69.1 | 69.1 |
| Smoking | 0.64(0.54–0.75) | 65.5 | 63.6 | 64.3 | 64.8 |  | 0.70(0.61–0.82) | 70.9 | 69.1 | 69.6 | 70.3 |
| Gensini score | 0.65(0.54–0.75) | 56.4 | 66.7 | 62.2 | 60.0 |  | − | − | − | − | − |
| Gender (male, n) | − | − | − | − | − |  | 0.69(0.59–0.79) | 98.2 | 40.0 | 62.1 | 95.7 |
| Logistic Regression Model ^#^ | 0.91(0.86–0.96) | 85.5 | 77.8 | 79.7 | 84.0 |  | 0.92(0.86–0.97) | 90.9 | 85.5 | 86.2 | 90.4 |

LVM: left ventricular mass; GLPS: global longitudinal peak strain; AUC: area under curve; MetS: metabolic syndrome; NT-proBNP: amino-terminal pro-B-type natriuretic peptide; PPV: positive predictive value; NPV: negative predictive value.

* NT-proBNP was log-transformed before being included in the regression analysis.

^#^ the model combined MetS, NT-proBNP, smoking, and Gensini score in GLPS; the model combined MetS, NT-proBNP, smoking, and Gender (male) in LVM.
