# Supplementary material for: The additive effect of metabolic syndrome on left ventricular impairment in patients with obstructive coronary artery disease assessed by 3.0 T cardiac magnetic resonance feature tracking
Source: Cardiovasc Diabetol. 2024 Apr 23;23:133. doi: 10.1186/s12933-024-02225-y (PMC11040951; doi:10.1186/s12933-024-02225-y)
Supplement: Supplementary file 2 — Additional file 2: Table S4. Inter- and intra-observer variability of CMR parameters. [file 12933_2024_2225_MOESM2_ESM.docx]

**Table S4 Inter- and intra-observer variability of CMR parameters**

|  | **Inter-observer (n = 40)** | |  | **Intra-observer (n = 40)** | |
| --- | --- | --- | --- | --- | --- |
|  | **ICC** | **95% CI** |  | **ICC** | **95% CI** |
| **Cardiac function parameters** | | | | | |
| LVEDV (ml) | 0.989 | 0.979-0.994 |  | 0.972 | 0.948-0.985 |
| LVESV (ml) | 0.996 | 0.992-0.998 |  | 0.993 | 0.987-0.996 |
| LVM (g) | 0.988 | 0.977-0.994 |  | 0.984 | 0.970-0.991 |
| **LV strain parameters** | | | | | |
| GRPS (%) | 0.982 | 0.967-0.991 |  | 0.980 | 0.963-0.989 |
| GCPS (%) | 0.966 | 0.937-0.982 |  | 0.985 | 0.972-0.992 |
| GLPS (%) | 0.948 | 0.903-0.972 |  | 0.966 | 0.936-0.982 |

Abbreviations as listed in Tables 1 and 2
